# Biofilm formation of mixed *Candida albicans* and methicillin-sensitive *Staphylococcus aureus* and surface properties of a 3D-printed denture base resin under different printing parameters

**DOI:** 10.1590/1678-7765-2025-0526

**Published:** 2026-01-19

**Authors:** Larianne S. Moisés, Hamile Emanuella do Carmo Viotto, Sabrina Romão Gonçalves Coelho, Danny Omar Mendoza Marin, Raphael Freitas Souza, Ana Carolina Pero

**Affiliations:** 1 Universidade Estadual Paulista Faculdade de Odontologia de Araraquara Departamento de Materiais Dentários e Prótese Araraquara SP Brasil Universidade Estadual Paulista (UNESP), Faculdade de Odontologia de Araraquara, Departamento de Materiais Dentários e Prótese, Araraquara, SP, Brasil.; 2 Universidade Federal de Santa Catarina Centro de Ciências da Saúde Departamento de Odontologia Florianópolis SC Brasil Universidade Federal de Santa Catarina, Centro de Ciências da Saúde, Departamento de Odontologia, Florianópolis, SC, Brasil.; 3 Université Laval Faculty of Dental Medicine Quebec Canada Université Laval, Faculty of Dental Medicine, Quebec, Canada.

**Keywords:** Denture bases, Biofilms, Printing, Three-Dimensional

## Abstract

**Objective:**

To evaluate the formation of mixed-species biofilms of *Candida albicans* and methicillin-sensitive *Staphylococcus aureus* (MSSA) on the surface of a 3D-printed denture base resin, as well as its surface properties, under varying printing parameters.

**Methodology:**

Discs (n=40 per group, 10×1.2 mm) of a denture base resin (priZma 3D Bio Denture) were fabricated using two 3D-printers—Liquid Crystal Display (LCD) and Digital Light Processing (DLP)—at three different angles (0°, 45°, or 90°). Surface roughness was measured using a digital profilometer and expressed as Ra (µm). For surface energy (SE) analysis, contact angles were measured using a tensiometer. Discs were incubated at 37 °C for 90 minutes and 48 hours to enable biofilm formation using *C. albicans* and MSSA inocula. Cell viability was assessed by colony-forming unit (CFU/mL) counts, and metabolic activity was evaluated using the XTT assay (absorbance). Microbial counts and XTT results were analyzed by three-way ANOVA (printer type, printing angle, incubation period). Surface roughness was analyzed by two-way ANOVA (printer type, printing angle), with Tukey’s test and a significance level of 0.05.

**Results:**

For both CFU/mL and XTT assays, incubation period was the only significant factor (p<0.001 and p=0.006, respectively), while other factors and interactions were not statistically significant (p>0.05). Surface roughness was significantly influenced by printer type, printing angle, and their interaction (p=0.027). The LCD 0° and LCD 90° groups produced smoother surfaces compared with LCD 45° (p=0.002), which showed similar values to all DLP groups regardless of angle (p>0.05). The DLP printer did not show significant roughness variations across the tested angles (p>0.05). The LCD groups presented numerically lower SE values compared to the DLP groups.

**Conclusion:**

The LCD system performs better than DLP in reducing surface roughness at 0° and 90°. Moreover, the analyzed factors did not significantly affect microbial adhesion or the formation of mixed-species biofilms.

## Graphical abstract

**Figure:**
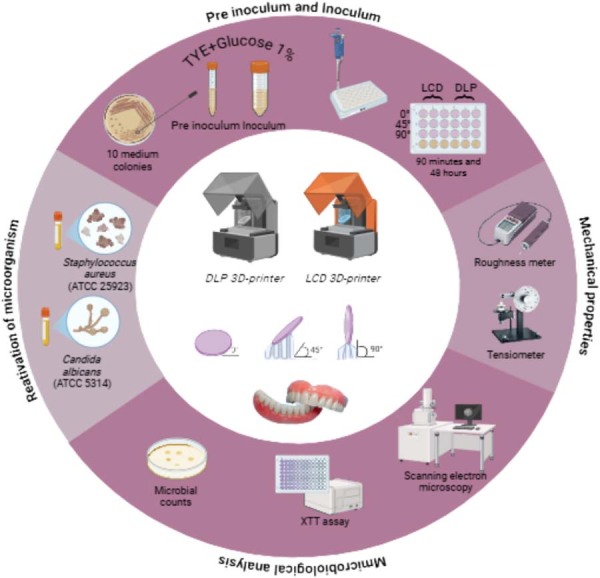

## Introduction

The digital additive technique (3D Printing) for manufacturing denture bases offers several advantages, including highly detailed reproduction of the components, time savings, and reduced material waste.^1-3^Different 3D printing systems have been proposed, including stereolithography (SLA), digital light processing (DLP), and liquid crystal display (LCD) systems. SLA 3D printing employs a laser beam (150–300 µm) that cures the resin by scanning each layer using a galvanometer system, providing high precision and excellent surface finish. However, the process is relatively slow, as the laser must scan the entire surface layer by layer. In contrast, the DLP system cures an entire layer at once using digital micromirror devices (DMDs), enabling faster printing speeds.^2,4^ A main disadvantage of the DLP system is that its print resolution depends on the density of micromirrors forming the DMD and the size of the projected surface.

The most recent advancement in 3D printing technology is the LCD system, which has transformed additive manufacturing due to its lower cost, high printing speed, and compatibility with the same resins used in DLP processes.^5,6^ LCD printers employ a liquid crystal display in which each pixel functions as a small window that either blocks or enables light to pass. This system can also filter light by adjusting the intensity of each pixel individually, achieving surface quality comparable to that of SLA.

The appropriate selection of printing parameters may influence the mechanical and biological properties of 3D-printed materials.^2,3^Parameters such as layer thickness, post-curing time, printing system, and printing angle can influence surface properties—including roughness, surface energy, and hydrophobicity/hydrophilicity—which in turn affect biofilm adhesion and formation.^7-12^

Acrylic resin denture bases are considered favorable environments for the proliferation and survival of oral microorganisms and microbial biofilm formation,^13^ due to the ease with which microbial cells adhere to surface roughness and pores present in the material. Microbial growth on denture surfaces occurs via the adhesion of microbial species to both the denture and oral mucosa, leading to a chronic inflammatory response known as denture stomatitis.^14,15^

*Candida albicans*, a commensal fungus, is the most prevalent species associated with denture stomatitis.^16^ One factor attributed to its virulence is its ability to interact with other microorganisms in the oral environment. *Staphylococcus aureus* (MSSA), a Gram-positive bacterium also frequently found in oral candidiasis, is particularly significant due to its high virulence and ability to develop resistance to drug therapies.^17^ Previous studies indicate that interactions between *C. albicans* and *S. aureus* in polymicrobial infections increase disease severity,^18^ such as denture stomatitis,^19^ and elevates the risk of systemic infections that are difficult to manage.^20^

The synergistic relationship between *C. albicans* and *S. aureus* within biofilms enhances microbial adhesion, structural complexity, and tolerance to antifungal and antibacterial agents. This cooperative behavior not only complicates eradication strategies but also contributes to persistent infections and treatment failures in clinical settings.^21^ The mechanisms of microbial adhesion to denture surfaces are associated with surface properties such as roughness and electrostatic interactions, including hydrophobic forces.^22^

Studies evaluating biofilm formation on 3D-printed denture base materials with varying printing parameters are still limited in the literature. Turanoglu, Talay Cevlik, and Vural^5^ (2024) evaluated the surface properties and adhesion of *Candida spp*. on a resin produced via LCD 3D printing and observed that specimens printed at 45º presented greater roughness, which did not result in greater adhesion of *Candida spp*.

Thus, this study aimed to evaluate the adhesion and formation of mixed-species biofilms of *C. albicans* and MSSA on the surface of a 3D-printed denture base resin, as well as to assess its surface properties, under different printing angles (0°, 45°, or 90°) and printing systems (DLP and LCD). The null hypothesis was that microbial viability on the resin surface would remain unaffected by the printing parameters (angles and systems), regardless of the incubation period (90 min or 48 h). Furthermore, it was hypothesized that the printing parameters do not exert a significant influence on either surface roughness or surface energy of the tested resin material.

## Methodology

Six experimental groups were established according to the printing angle (0º, 45º, or 90º) and printing system (DLP and LCD). A total of 240 circular specimens measuring 10 mm in diameter and 1.2 mm in thickness were fabricated using the denture base resin priZma 3D Bio Denture (Makertech Labs, SP, Brazil), with 40 specimens prepared per group. Microbiological tests were performed on three independent occasions, in triplicate, for both 90 min (adhesion) and 48 h (biofilm). For each occasion and each period, six specimens per group were used for CFU/mL (n=3) and XTT (n=3). Thus, for microbiological assays (CFU/mL and XTT), a total of 36 specimens per group was used. Additionally, two specimens per group were produced for scanning electron microscopy (SEM) and two specimen per group for surface energy (SE) analysis.

Specimens were fabricated using the LCD printing system with the Flashforge Foto 6.0 2K printer (Zhejiang Flashforge3D Technology Co., Jinhua City, Zhejiang, China) and the DLP 3D printer Flashforge Hunter (Zhejiang Flashforge3D Technology Co., Jinhua City, Zhejiang, China). The virtual design of the specimens was created using Adobe Meshmixer v. 3.5 software (Autodesk Inc., San Rafael, CA, USA) and subsequently converted into an .stl file. This file was then imported into FlashDLPrint software v. 3.28.0 to define the print orientation at 0°, 45°, or 90°.

For the 0° orientation, specimens were printed lying flat on the build platform without support structures. The print layers were oriented along the height of the specimen on the Z-axis. For the 45° orientation, specimens were positioned at a 45° angle relative to the platform, with the layers also oriented at 45° along the Z-axis; in this case, support structures were required. For the 90° (vertical) orientation, specimens were positioned perpendicular to the build platform, with the print layers oriented along the length of the specimen on the Z-axis, and no support structures were used.

The polymerization process of the specimens on the printer platform was initiated by ultraviolet light activation (LED panel, λ=405 nm). Specimen printing was conducted according to the manufacturer’s instructions, using a layer thickness of 50 µm. In this process, the first four layers underwent over-curing for 20 seconds each, while subsequent layers were cured for 3 seconds each at 80% light intensity to slow down polymerization and reduce polymerization shrinkage.

After printing, specimens were washed in 99% isopropyl alcohol for 5 minutes to remove residual monomer from their surfaces,^23^ then immersed in liquid glycerin and subjected to post-curing with UV light at a wavelength of 405 nm in a Form Cure unit (Formlabs, Inc., Somerville, MA, USA) for 10 minutes, following the resin manufacturer’s recommendations—5 minutes on each side.^8^

Prior to testing, specimens were cleaned for 1 minute in an ultrasonic bath containing distilled water with 1% detergent and dried using absorbent paper. Subsequently, they were stored in distilled water at 37°C for 50±2 hours.

Surface roughness was evaluated using a portable digital profilometer (SJ-400; Mitutoyo Corp.) with a resolution of 0.01 µm, a measurement length of 2.4 mm, a stylus speed of 0.5 mm/s, and a stylus tip radius of 5 µm. Two measurements were taken in the central region of each specimen, and the average roughness (Ra, µm) was calculated for each experimental condition. The Ra value corresponds to the arithmetic average of the absolute deviations from the mean line of the surface profile. All measurements were performed by a single trained operator.^24^

For SE analysis, contact angle measurements were performed using three liquids (water, formamide, and diiodomethane) at a controlled temperature of 25 °C, employing a tensiometer (DATAPHYSICS Instruments GmbH Contact Angle System OCA20, Filderstadt, Germany). The contact angle was measured using the sessile drop method, in which 10 µL of each liquid were dispensed onto the sample surface. Contact angles were automatically calculated by fitting the droplet shape captured after 10 seconds (SCA20 software), according to the Young–Laplace equation. The SE of the sample coating was calculated using the Owens–Wendt–Rabel–Kaelble (OWRK) method to determine dispersive and polar components. Data were analyzed descriptively. Two specimens were evaluated per group.

Each specimen was placed on the device platform, positioned between the camera and the lens. A consistent droplet volume of each liquid was used for all measurements, and a 10-second wait time was allowed for liquid stabilization and interaction with the surface before recording. The right and left contact angles between the specimen surface and each droplet were measured, and the final contact angle was calculated using the Young–Laplace equation.^25^ These values were then used to calculate the SE based on the polar and dispersive components using the OWRK method.^26^ Two measurements were performed on distinct areas of each specimen, and the arithmetic mean was calculated for each experimental condition. All measurements were conducted by a single operator.

The microbial strains used (*C. albicans* SC5314 and *Staphylococcus aureus* ATCC 25923) were stored frozen at −80°C and reactivated at the time of use. Reactivation was performed on agar plates containing SDA supplemented with chloramphenicol for *C. albicans* and BHI agar supplemented with amphotericin B for *S. aureus*, using the streak plate method. The plates were then incubated at 37°C for 48 hours. After incubation, a defined number of colonies from each strain was separately transferred into a Tryptone and Yeast Extract (TYE) broth supplemented with 1% glucose and incubated in a shaker incubator for 16 hours (*C. albicans*) and 18 hours (*S. aureus*) to obtain the pre-inoculum. Following incubation, microbial concentrations were adjusted by diluting the cultures in TYE + 1% glucose medium at 1:10 for *C. albicans* and 1:20 for *S. aureus*. Microorganisms were maintained in the exponential growth phase (mid-log) by measuring the optical density (OD) in a spectrophotometer, ensuring a final concentration of 1 × 10⁷ CFU/mL.

Mixed biofilms of *C. albicans* and *S. aureus* underwent the adhesion phase, which consisted of incubation under orbital agitation at 75 rpm and 37°C for 90 minutes. After the adhesion phase, all discs were carefully removed from the plates and washed with 1X PBS to remove non-adherent cells. Discs designated for adhesion-phase analysis were randomly selected and transferred to sterile plates for sonication and scraping.^27^

For the remaining discs intended for 48-hour biofilm evaluation, 1.5 mL of fresh TYE medium supplemented with 1% sucrose was added to each well containing the adherent microorganisms. The plates were then incubated under orbital agitation at 37°C for 48 hours to enable mature biofilm formation. To assess both the adhesion phase and the 48-hour biofilm, the discs were transferred to sterile plates containing 1.5 mL of sterile saline solution and placed in an ultrasonic bath for 10 minutes. After this period, the biofilms were mechanically removed using a sterile spatula. The collected biofilms were transferred to Eppendorf tubes and subjected to serial dilution. Dilutions were plated on agar media (*C. albicans* on SDA + chloramphenicol, and *S. aureus* on BHI + amphotericin B) and incubated at 37°C for 48 hours. Following incubation, colony-forming units (CFUs) were counted for both periods.

Afterward, specimens containing biofilms were processed in the same manner described for the adhesion assays.

The number of colony-forming units per milliliter (CFU/mL) was calculated using the following formula:


$$\frac{\text{ CFU }}{\mathrm{mL}}=\frac{\text{ number of colonies }\times 10^{n}}{q}$$

In which *n* corresponds to the absolute value of the dilution (1, 2, 3, 4, 5, 6, or 7) and *q* represents the volume, in milliliters, pipetted for each dilution during plate seeding. CFU/mL values were expressed in scientific notation, and the arithmetic mean of the triplicates for each sample was calculated. The resulting data were transformed using the formula: log(CFU+1)/mL.

Cellular metabolism was assessed using the XTT assay. XTT solution (2,3-bis (2-methoxy-4-nitro-5-sulfophenly) - 5 - [(phenylamino) carbonyl] - 2H - tetrazolium hydroxide) was prepared at 2.5 mg/mL with 0.4 μm menadione and stored at −70°C until use. After biofilm formation (adhesion phase and 48 hours), the discs were transferred to PBS, biofilms were scraped, and 100 μL of the resulting suspension was placed in a 96-well plate.^27^ A reaction mixture containing PBS with glucose, XTT, and menadione was added, and the plates were incubated in the dark at 37°C for t hours. Absorbance was measured at 492 nm using a microplate reader, with the XTT solution as the blank.

After the adhesion phase and mature biofilm formation on the specimens, they were washed with PBS to remove non-adherent cells and then subjected to a fixation and progressive dehydration for scanning SEM analysis. The biofilm adhered to the specimens was fixed by immersion in 2.5% glutaraldehyde for one hour at room temperature. Following fixation, the specimens were washed three times with PBS and then progressively dehydrated in ethanol solutions: 70% ethanol (1 hour), 90% ethanol (1 hour), and absolute ethanol (five washes of 30 minutes each). After dehydration, the specimens were placed in a vacuum desiccator with silica gel for one week. Next, the specimens were carbon-coated (Denton Vacuum, Moorestown, New Jersey, USA) and examined using a scanning electron microscope (JEOL JSM-6610LV, Akishima, Japan) at a magnification of 500x. This procedure was performed in duplicate on a single occasion.

The sample size was determined based on previous studies using the same dependent variables.^27^ Microbial count data were log10-transformed prior to analysis. Results were expressed as means and standard deviations (SD) and tabulated according to experimental conditions. Microbial counts and XTT assay results were analyzed using a three-way ANOVA, with printer type, printing angle, and incubation period as independent factors. Surface roughness was analyzed using a two-way ANOVA, considering printer type and printing angle. Post hoc comparisons were performed using Tukey’s HSD test. A significance level of 0.05 was adopted for all analyses, which were conducted using IBM SPSS Statistics, v. 29.

## Results

Table 1 presents descriptive statistics for the log10-transformed microbial counts of *C. albicans* and *S. aureus*, and Table 2 shows the XTT assay results. Microbial counts varied notably across different incubation periods. This trend was confirmed by the three-way ANOVA for *C. albicans* (Table 3), in which incubation period was a significant factor (p<0.001), while printer type, printing angle, and their interactions were not statistically significant (p>0.05). Table 4 shows that incubation period was the only significant factor for *S. aureus* and XTT results (p<0.001 and p=0.006, respectively), with all other sources of variation and interactions remaining non-significant (p>0.05).

**Table 1:** Mean (SD) log10-transformed microbial counts after 90 min and 48 h of incubation.

			Results	
Variable	Printer type	Angle, degrees	90 min	48 h
*C. albicans*	LCD	0	6.3 (0.1)	7.2 (0.1)
		45	6.3 (0.1)	7.3 (0.1)
		90	6.3 (0.2)	7.1 (0.4)
	DLP	0	6.4 (0.2)	7.2 (0.1)
		45	6.3 (0.1)	7.2 (0.1)
		90	6.4 (0.2)	7.2 (0.0)
*S. aureus*	LCD	0	6.6 (0.3)	7.8 (0.0)
		45	6.5 (0.2)	7.8 (0.1)
		90	6.6 (0.2)	7.8 (0.1)
	DLP	0	6.5 (0.3)	7.8 (0.1)
		45	6.5 (0.3)	7.8 (0.0)
		90	6.5 (0.3)	7.8 (0.1)

**Table 2:** XTT results (absorbance at 492 nm) after 90 min and 48 h of incubation.

			Results	
Variable	Printer type	Angle, degrees	90 min	48 h
XTT	LCD	0	2.2 (0.2)	2.4 (0.1)
		45	2.0 (0.3)	2.3 (0.2)
		90	2.0 (0.2)	2.0 (0.5)
	DLP	0	2.2 (0.2)	2.3 (0.3)
		45	2.2 (0.3)	2.2 (0.2)
		90	2.0 (0.5)	2.3 (0.3)

**Table 3:** Three-way ANOVA results for log10-transformed microbial counts.

Variable	Source	SS	df	MS	F	p-value
*C.albicans*	Printer	0.043	1	0.043	1.457	0.23
	Angle	0.046	2	0.023	0.788	0.458
	Period	19.126	1	19.126	652.584	<0.001 *
	Printer x Angle	0.093	2	0.047	1.591	0.209
	Printer x Period	0.01	1	0.01	0.354	0.553
	Angle x Period	0.18	2	0.09	3.075	0.051
	Printer x Angle x Period	0.001	2	0.001	0.02	0.98
	Error	2.814	96	0.029		
	Total	22.313	107			
*S. aureus*	Printer	0.003	1	0.003	0.095	0.759
	Angle	0.033	2	0.016	0.499	0.609
	Period	41.676	1	41.676	1.269.929	<0.001 *
	Printer x Angle	0.004	2	0.002	0.06	0.942
	Printer x Period	0.001	1	0.001	0.033	0.856
	Angle x Period	0.008	2	0.004	0.119	0.888
	Printer x Angle x Period	0.007	2	0.003	0.103	0.902
	Error	3.151	96	0.033		
	Total	44.882	107			

* Statistically significant, p<0.05.

**Table 4:** Three-way ANOVA results for XTT.

Variable	Source	SS	df	MS	F	p-value
XTT	Printer	0.087	1	0.087	0.93	0.337
	Angle	0.554	2	0.277	2.958	0.057
	Period	0.753	1	0.753	8.035	0.006 *
	Printer x Angle	0.177	2	0.089	0.946	0.392
	Printer x Period	0.005	1	0.005	0.058	0.81
	Angle x Period	0.006	2	0.003	0.031	0.97
	Printer x Angle x Period	0.483	2	0.241	2.576	0.081
	Error	8.991	96	0.094		
	Total	11.056	107			

* Statistically significant, p<0.05.

Table 5 presents the surface roughness measurements of the tested specimens. Two-way ANOVA revealed that both printer type and printing angle significantly influenced roughness (both p<0.001), as did their interaction (p=0.027). Post hoc comparisons indicated that the LCD 0° and LCD 90° groups produced smoother surfaces compared with LCD 45° (p=0.002), which was similar to DLP, regardless of printing angle (p>0.05). The DLP printer did not show significant variations in roughness across the tested angles (p>0.05).

**Table 5:** Mean (SD) roughness in Ra (µm). Similar uppercase letters represent non-significant differences between different printing systems and printing angles (Tukey’s HSD test, p<0.05).

Angle, degrees	LCD	DLP
0	1.80 (0.28)^A^	2.58 (0.39)^B^
45	2.59 (1.12)^B^	2.90 (0.78)^B^
90	1.61 (0.51)^A^	2.78 (0.57)^B^

Table 6 presents the contact angle measurements obtained for each experimental group, according to the liquid used.

**Table 6:** Mean (SD) contact angles for each experimental condition.

Angle, degrees	LCD			DLP		
	Water	Formamide	Diiodomethane	Water	Formamide	Diiodomethane
0	90.38 (8.10)	64.66 (10.64)	51.86 (3.91)	89.72 (14.56)	66.52 (19.03)	35.30 (6.60)
45	79.14 (2.79)	69.28 (5.56)	47.64 (2.46)	96.54 (9.33)	72.98 (5.53)	41.76 (6.32)
90	78.80 (12.49)	68.78 (4.00)	43.96 (2.62)	87.52 (3.74)	73.30 (4.82)	40.90 (8.37)

**Figure 1:**
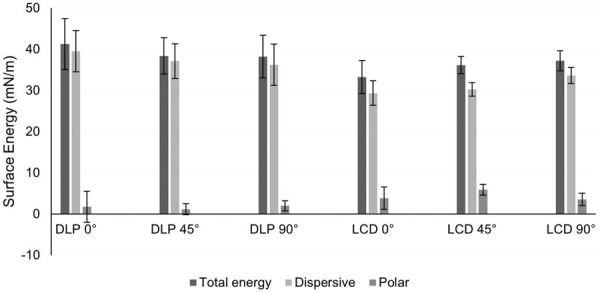
Mean values and standard deviations of total, dispersive, and polar surface energy (SE) for each experimental group. Descriptive data.

Figure 1 shows a descriptive analysis of SE (mN/m) for each experimental condition. For each group, the bars represent the total SE, dispersive SE, and polar SE in mN/m. Total surface energy expresses all attractive forces between molecules, predicting the potential for chemical and physical interactions with other substances. Dispersive surface energy is a component of the total SE, even though they are a weak and undulating ingredient, and polar surface energy is the component of SE that results from the attraction of charges between molecules that have areas permanently charged, either positively or negatively. The DLP groups showed numerically higher total SE than the LCD groups, regardless of printing angle. These measurements were performed solely to characterize the samples and support the discussion, as in previous studies.^28,29^

**Figure 2:**
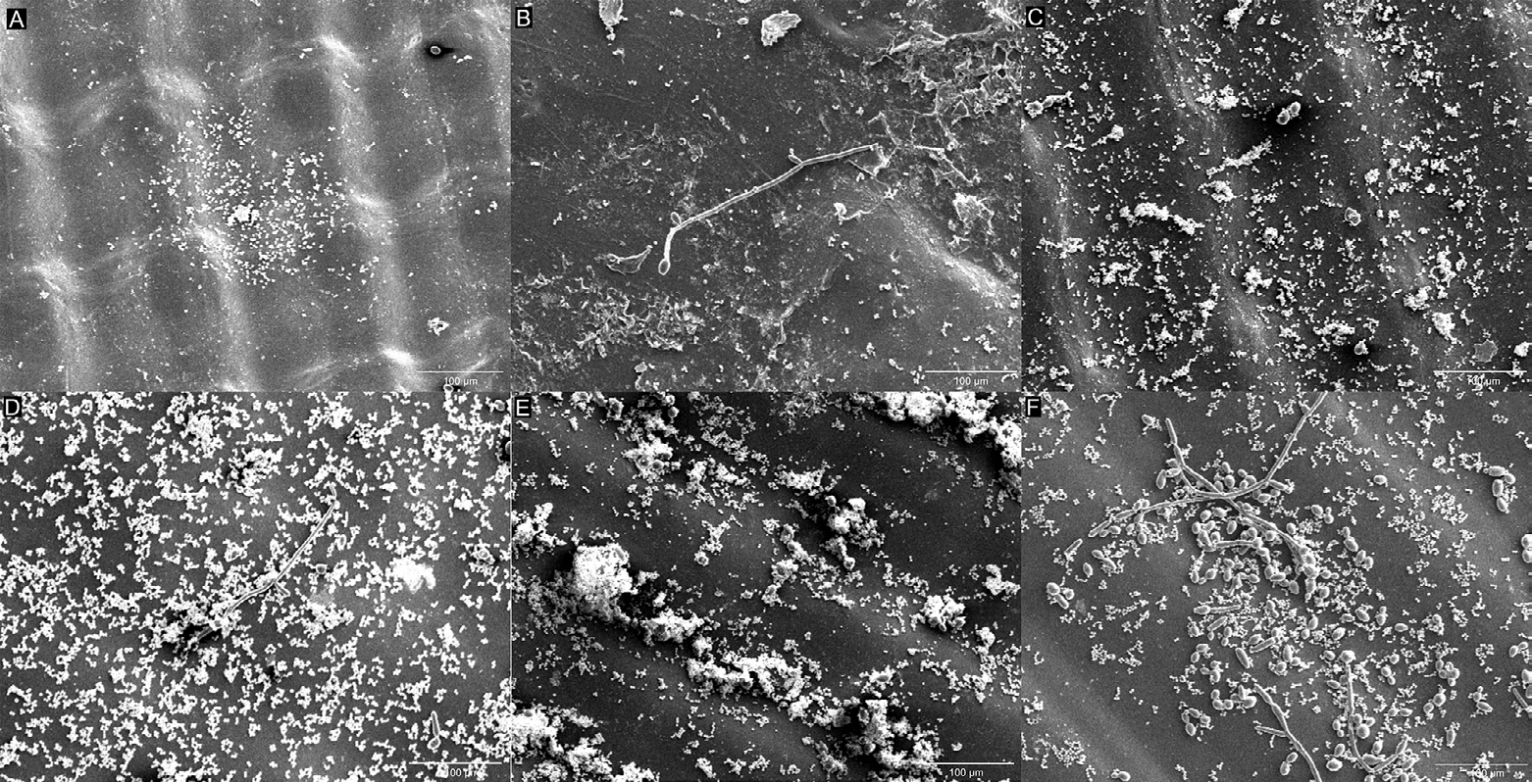
Scanning Electron Microscopy (SEM) images of adhesion and mature biofilm on LCD specimens at 500× magnification: A, 90 minutes at 0°; B, 48 hours at 0°; C, 90 minutes at 45°; D, 48 hours at 45°; E, 90 minutes at 90°; F, 48 hours at 90°.

**Figure 3:**
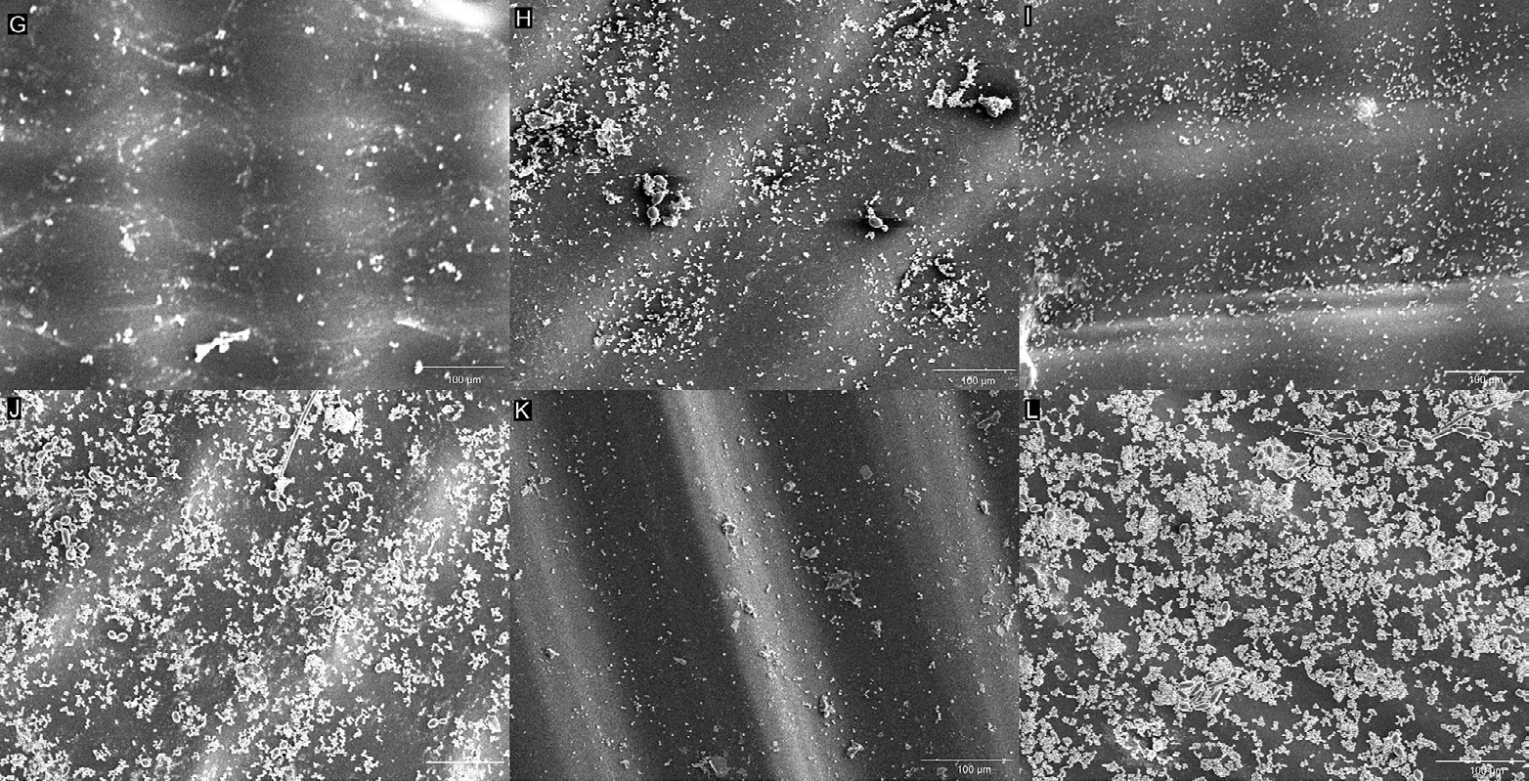
Scanning Electron Microscopy (SEM) images of adhesion and mature biofilm on DLP specimens at 500× magnification: G, 90 minutes at 0°; H, 48 hours at 0°; I, 90 minutes at 45°; J, 48 hours at 45°; K, 90 minutes at 90°; L, 48 hours at 90°.

The SEM analysis revealed visible morphological changes between the initial adhesion phase and mature biofilm formation. Figures 2A, 2C, and 2E for the LCD groups and Figures 3G, 3I, and 3K for the DLP groups show the prevalence of yeast and cocci-shaped cells during the initial adhesion phase (90 min). In contrast, Figures 2B, 2D, and 2F for the LCD groups and Figures 3H, 3J, 3L for the DLP groups display pseudohyphae and short hyphae in the mature biofilm after 48 h. The images indicate that specimens fabricated using the LCD system exhibited a more robust biofilm adhered to their surfaces.

## Discussion

The null hypothesis was partially accepted, as microbial viability on the resin surface was not affected by the printing angle, regardless of the incubation period (90 minutes or 48 hours). However, both printer type and printing angle significantly influenced surface roughness (both p<0.001). Roughness was higher in specimens printed at 45° using the DLP system and similar to LCD, while those printed at 0° and 90° showed smoother surfaces.

Printing orientation determines the direction of the layers, affecting porosity, morphological features, and surface geometry of printed objects, making 3D printing highly sensitive to these parameters.^3,24^ Al-Dulaijan, et al.^11^(2022) compared surface roughness of 3D-printed denture base materials with heat-polymerized resin and reported increased roughness at 45° and 90° compared with 0°. Shim, et al.^23^ (2020) also indicated that samples printed at 45° showed rougher surfaces due to the gradual addition of layers, resulting in stepped edges between layers and increased surface roughness. Turanoglu, Talay Cevlik, and Vural^5^ observed under microscopy that LCD-printed specimens at 45º presented rectangular patterns formed by horizontal and vertical lines, whereas Li, et al.^9^ (2022) demonstrated that DLP-printed specimens at 45º exhibited a stepwise pattern. Thus, variations in roughness between printing angles have been attributed to layer arrangement and gaps between them.^5,11,23,27^

Despite the higher roughness observed in the DLP printing system, mixed biofilms formed on its surface exhibited similar cell viability (CFU/mL) and XTT compared to the LCD printing system. Notably, several studies report divergent behaviors regarding microbial adhesion.^10,26,27^ Turanoglu, Talay Cevlik, and Vural^5^(2024) evaluated the effect of printing angle using the LCD system on surface properties and *Candida* adhesion; however, the resin in their study was specific for surgical guides. They found that specimens printed at 45° exhibited higher roughness, which did not result in increased *Candida* adhesion. Shim, et al.^23^(2020) observed that denture base specimens with lower roughness, printed at 0°, showed greater *C. albicans* biofilm adhesion after 24 hours.

Although not yet fully understood, the mechanisms of microbial adherence to prosthetic surfaces are associated with surface properties such as roughness and electrostatic interactions, including hydrophobic forces.^22^ These substrate surface factors, along with environmental conditions, influence virulence traits of biofilms, which can be quantified by analyzing the expression of specific proteins. Due to the anisotropic behavior of 3D-printed resins, specific printing conditions—such as layer thickness, post-curing time, and printing angle—may influence the material’s surface properties, including roughness, surface energy, and hydrophobicity/hydrophilicity, thereby affecting microbial adhesion and biofilm formation.^7-10, 20,23,27^

Considering that roughness and surface energy did not affect microbial adhesion and biofilm formation on the denture base materials obtained in this study, it can be hypothesized that other factors—such as surface charge,^30^ hydrophobicity,^31-33^ chemical composition of the resin,^35,36^ and interactions among microorganisms^36^—may have influenced microbiological results. Surface roughness influences initial microbial adherence, as irregular surfaces retain microorganisms and protect them from mechanical forces during denture cleaning and salivary factors.^36,37^The threshold mean roughness (Ra) for bacterial adhesion is 0.2 μm.^38^Regarding SE, higher SE values also contribute to increased microbial adhesion, with values above 50 mJ/m^2^ being critical for microorganisms to adhere via glycoprotein pellicle precursor formation.^9,24^

The results of this study demonstrated that SE was also influenced by the printing parameters. Although the SE values did not reach the critical threshold (50 mJ/m^2^), numerically higher values were observed for the DLP groups. Within the same printing system, high SE appeared at 0° for DLP and 90° for LCD. However, these results did not statistically influence microbial adhesion. Shim, et al.^23^(2020) also observed that *C. albicans* adhesion was not influenced by SE, since the greatest adhesion occurred at 0° and the highest SE value was at 45°.

The SEM analysis revealed a predominant yeast morphology for *C. albicans* and cocci for *S. aureus*. The polymorphism of *C. albicans*—including yeast, pseudohyphae, and hyphal forms—is a key virulence factor. Among these, the hyphal form is particularly pathogenic due to its ability to exert mechanical force, penetrate host epithelial tissue, cause endothelial damage, and facilitate dissemination of infection via the bloodstream.^39^

However, in this study, the predominance of yeast forms may be related to the *in vitro* biofilm development model. The data suggest that during biofilm formation, cells may continuously disperse, remaining mostly, if not exclusively, in the spherical yeast form. Although these dispersed cells morphologically resemble the rounded yeast cells found in planktonic growth, they exhibit distinct characteristics.^40^

The findings of this study indicate that the LCD system is a viable alternative for 3D printing denture bases, showing lower surface roughness at all tested angles compared to the DLP system. Additionally, mixed biofilm adhesion was statistically similar between both systems. Considering these favorable results, along with its low cost, high printing speed, and compatibility with resins used in DLP systems, the LCD system represents an efficient and cost-effective option for additive manufacturing of denture bases. The literature on LCD-printed denture bases is still limited. Furthermore, the results of this study are consistent with Li, et al.^9^ (2022), who also concluded that the LCD system may represent an affordable, faster, and promising system for printing denture bases, demonstrating performance comparable to SLA and DLP systems. This highlights the relevance of the results obtained in this study.

The limitations of this study include the evaluation of only one brand of 3D-printed denture base resin and the use of only two microorganisms in the mixed biofilm model. The use of only two specimens for SE analysis precluded statistical analysis, which is another limitation. Specimen shape differed from that of actual denture bases, and the surfaces may not fully represent clinical conditions due to the complex geometry of dentures. Additionally, the metabolic activity of the scrapped biofilm may not accurately represent the metabolism of cells organized within a biofilm. Further optimization of printing parameters may help improve surface roughness. Future studies addressing these factors could help clarify the findings.

## Conclusions

Considering the limitations of this *in vitro* study, it can be concluded that printing angles and systems did not significantly affect microbial adhesion or formation of mixed-species biofilms on the denture base resin, despite increased surface roughness at 45° for both systems (DLP and LCD). Thus, the LCD system represents an efficient and cost-effective alternative for additive manufacturing of denture bases.
